# Histological confinement of transglutaminase-mediated nit sheath crosslinking is essential for proper oviposition and egg coating in the human head louse, *Pediculus humanus capitis*

**DOI:** 10.1186/s13071-023-05720-5

**Published:** 2023-03-09

**Authors:** Ju Hyeon Kim, Do Eun Lee, Sang Youn Park, John M. Clark, Si Hyeock Lee

**Affiliations:** 1 Department of Tropical Medicine and Parasitology, Seoul National University College of Medicine, Seoul, 03080 Republic of Korea; 2 Institute of Endemic Diseases, Seoul National University College of Medicine, Seoul, 03080 Republic of Korea; 3 Department of Agricultural Biotechnology, Seoul National University, Seoul, 08826 Republic of Korea; 4 School of Systems Biomedical Science, Soongsil University, Seoul, 06978 Republic of Korea; 5 Department of Veterinary and Animal Sciences, University of Massachusetts, Amherst, MA 01003 USA; 6 Research Institute of Agriculture and Life Sciences, Seoul National University, Seoul, 08826 Republic of Korea

**Keywords:** Head louse, Nit, Egg sheath, LNSP, Transglutaminase, Oviposition, *Pediculus humanus capitis*

## Abstract

**Background:**

Head louse females secrete liquid gel, which is mainly composed of the louse nit sheath protein 1 (LNSP1) and LNSP2, when they lay eggs. The gel is crosslinked by transglutaminase (TG) to form the nit sheath, which covers most of the egg except the top operculum area where breathing holes are located. Knowledge on the selective mechanism of nit sheath solidification to avoid uncontrolled crosslinking could lead to designing a novel method of louse control, but no information is available yet.

**Methods:**

To elucidate the crosslinking mechanisms of nit sheath gel inside the reproductive system of head louse females, in situ hybridization in conjunction with microscopic observation of the oviposition process was conducted.

**Results:**

Histochemical analysis revealed that *LNSP1* and *LNSP2* are expressed over the entire area of the accessory gland and uterus, whereas TG expression site is confined to a highly localized area around the opening of posterior oviduct. Detailed microscopic observations of oviposition process uncovered that a mature egg is positioned in the uterus after ovulation. Once aligned inside the uterus, the mature egg is redirected so that its operculum side is tightly held by the ventral end of the uterus being positioned toward the head again and its pointed bottom end being positioned toward the dorsal end of the uterus, which functions as a reservoir for the nit sheath gel.

**Conclusions:**

Physical separation of the TG-mediated crosslinking site from the ventral end of the uterus is necessary to avoid uncontrolled crosslinking inside the uterus and to ensure selective crosslinking over only the lower part of egg without any unwanted crosslinking over the operculum during oviposition.

**Graphical Abstract:**

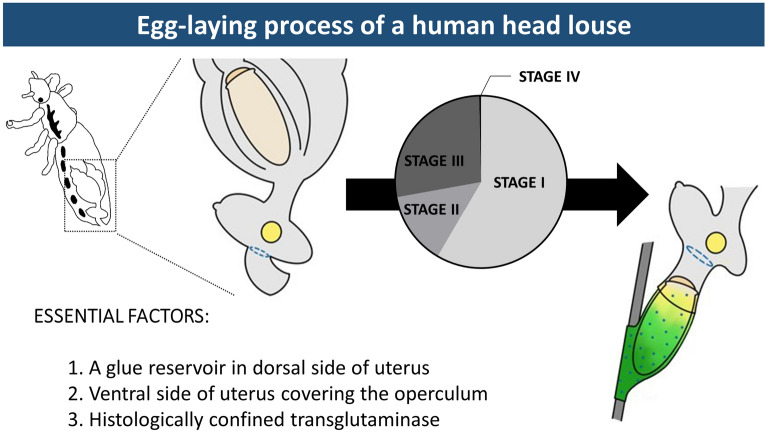

**Supplementary Information:**

The online version contains supplementary material available at 10.1186/s13071-023-05720-5.

## Background

Both head lice (*Pediculus humanus capitis*) and body lice (*P. humanus humanus*) are obligatory ectoparasites, exclusively feeding on human blood. Human head lice cause economic and social problems worldwide, whereas human body lice pose serious public health threats by transmitting several bacterial diseases [1]. Human louse females secrete liquid gel (louse glue), which later forms a protective egg covering, called the nit sheath, to attach newly laid eggs to hair or fabrics. The nit sheath gel is produced from a pair of large female accessory glands and secreted into the uterus. For oviposition, a female louse secretes the gel onto the hair shaft first, spreads it using the last abdominal segment and then lays an egg, with the pointed (bottom) end of the egg being released first along with the gel [2]. The gel secretion diminishes before the anterior operculum side of the egg is excluded, which likely contributes to the protection of the operculum, a lid-like structure where aeropyles for gas exchange are located and through which the first instar nymph hatches, from being occluded by the liquid gel [3]. Following oviposition, the nit sheath gel coated over mostly the bottom part of the egg is completely solidified to form the nit sheath, thereby attaching it to the hair [4].

By analyzing the amino acid composition of the nit sheath proteins, two homologous proteins, named louse nit sheath protein (LNSP) 1 and LNSP2, were identified [5]. More recently, LNSP1 and LNSP2 along with two hypothetical proteins were confirmed to be the major structural proteins by analyzing the transcriptome of the accessory gland plus uterus [6]. Using RNA interference (RNAi), LNSP1 and LNSP2 were determined to function in desiccation resistance and lubrication, thereby ensuring normal oviposition and embryo survival. In addition to LNSP1 and LNSP2, several proteins including transglutaminase (TG), defensin 1 and defensin 2 were identified to have essential functions. Knockdown of *TG* also impaired egg hatching, suggesting its role in the crosslinking of nit sheath protein. The role of TG in crosslinking was further confirmed by the treatment of GGsTop, a TG inhibitor. Taken together, controlled crosslinking of nit sheath gel, mainly composed of LNSP1 and LNSP2, is essential for producing a functional nit sheath that ensures egg viability besides its function as glue.

Uncontrolled rapid solidification of the nit sheath gel before the complete exclusion of an egg can be fatal to a female as the incompletely excluded egg sticks to the vagina and blocks oviposition. Sometimes, the female ovipositor is observed to be strongly stuck to a hair by abnormally solidified nit sheath gel, resulting in eventual death of the female during oviposition, as described previously [3]. This phenomenon was particularly observed in old females (JHK, personal observation). On the other hand, retarded solidification is also detrimental to egg development, as previously demonstrated by impairing the nit sheath gel crosslinking via GGsTop [6]. Since both LNSP1 and LNSP2 are exclusively expressed in the accessory gland [6], it is necessary to precisely regulate the crosslinking of nit sheath gel to occur not inside the accessory glands but inside the uterus only when a mature egg is housed. In addition, a specific mechanism is required to prevent the nit sheath covering and solidification over the operculum area. However, it is unknown yet how human lice avoid the uncontrolled crosslinking inside the reproductive system and regulate selective crosslinking of the nit sheath only over the bottom part of the egg.

In this study, therefore, to elucidate the crosslinking mechanisms of nit sheath gel inside the female reproductive system, in situ hybridization in conjunction with microscopic observation of the oviposition process was conducted. The detailed oviposition processes were constituted from multiple observations of dissected gravid females. A sophisticated mechanism for avoiding uncontrolled crosslinking inside the uterus and the selective crosslinking over only the lower part of the egg during oviposition was proposed.

## Methods

### Lice rearing

The South Florida strain of human head lice (SF-HL. *P. humanus capitis*) were reared on the in vitro membrane feeding system [7] under environmental conditions of 30 °C, 70% relative humidity and 16/8 h light/dark in a rearing chamber (reviewed and approved by the Institutional Review Board of Seoul National University, IRB no. E1911/003–016, E-2205-080-1324).

### Probe synthesis

Total RNA was extracted from 5-day-old female lice with TRI reagent (MRC, Cincinnati, OH, USA) and treated with DNase I (Takara Biotechnology, Shiga, Japan) according to the manufacturer's protocol. First-strand cDNA was synthesized using SuperScript IV reverse transcriptase (Invitrogen, Carlsbad, CA, USA).

For probe synthesis, *LNSP1*, *LNSP2* and *TG* fragments were amplified from the female cDNA (the primer sequences are shown in Additional file 1: Table S1). For *LNSP1* and *LNSP2*, which show high sequence similarities, respective probes were designed from gene-specific sites in the N terminal domains. The PCR products were cloned into pGEM-T Easy Vector (Promega, Madison, WI, USA). Each plasmid was digested by ApaI (New England Biolabs, Ipswich, MA, USA) for sense probe (negative control) or NdeI (New England Biolabs) for antisense probe at 37 °C for 1 h. The plasmids were checked by electrophoresis to confirm digestion and purified using Monarch® PCR & DNA Cleanup Kit (New England Biolabs). Sense or antisense *LNSP1*, *LNSP2* and *TG* probes were generated using T7 or SP6 RNA polymerase (Promega). FITC RNA labeling mix or DIG RNA labeling mix (both from Roche, Mannheim, Germany) were used for *LNSP1*/*LNSP2* probe or *TG* probes, respectively.

### In situ hybridization

Accessory glands and uterus were dissected from 5-day-old females in ice-cold RNase-free PBS (pH 7.4) (for reproductive system of human head louse, see additional file 2: Fig. S1). Tissues were incubated in 0.01% collagenase (Sigma) in PBS for 1 min with gentle rocking to improve the penetration of probe and reagents. After washing three times with PBS, tissues were fixed in 4% paraformaldehyde at 4 °C overnight. Tissues were washed with PBS, followed by dehydration and rehydration using a series of ethanol baths. Hybridization was conducted in hybridization solution containing *LNSP1*, *LNSP2* or *TG* probes for 20 h at 58 °C. After washing three times each with 5× and 0.2× SSC buffers at 63 °C, subsequent protocols were adjusted depending on the experiments. Tissues for *LNSP1* and *LNSP2* were washed with PBST and then mounted on a glass slide with Vectashield (Vector Laboratories, Burlingame, CA, USA) for further confocal microscopy (SP8 × STED confocal microscope, Leica). For the *TG* experiment, tissues were incubated with anti-DIG-AP, Fab fragments (Roche) in a blocking reagent at 4 °C overnight. The hybridization signal was visualized by treatment of NBT and BCIP (Roche) in NTMT buffer.

### Examination of oviposition process

The abdomens of 5-day-old gravid females were dissected in PBS under a stereo microscope (Greenough Stereo Microscopes S9i, Leica), and the oviposition stage of each dissected female was determined. Based on the observation that a 5-day-old female lays approximately five eggs a day, the time required for a single egg's oviposition was assumed to be ~ 290 min. After dissecting a total of 75 females, the duration of each stage was roughly estimated by the following calculation: (total number of observed oviposition stage/75) × 290 (min).

## Results

### Histological characterization of TG-mediated crosslinking inside the uterus

**Fig. 1:**
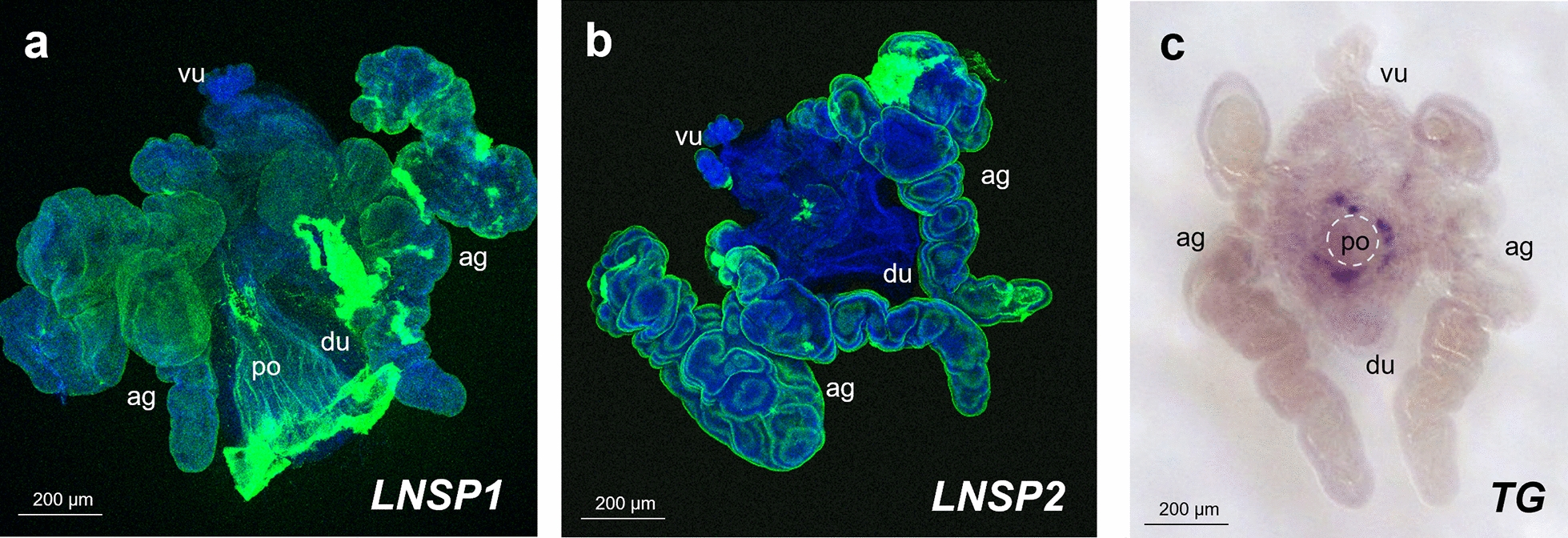
Histological sites of *LNSP1*, *LNSP2* or *TG* transcription in the accessory glands and uterus. Representative microscopic images of the accessory glands and uterus of head louse females following in situ hybridization with *LNSP1* (**a**), *LNSP2* (**b**) or *TG* (**c**) probe. **a**, **b** FITC-labeled probes were used for *LNSP1* and *LNSP2*. Both *LNSP1* and *LNSP2* were mainly detected in the entire areas of accessory gland with reduced expression being detected in the uterus (green signal). Images of nuclear staining (blue signal) and *LNSP1* or *LNSP2* were merged. **c** For *TG*, DIG-labeled probes were used for signal amplification. *TG* was exclusively expressed at the focal area around the opening of posterior oviduct (dark purple). A dotted circle indicates the opening of posterior oviduct where an egg passes through for oviposition. *ag* accessory gland, *vu* ventral end of uterus, *du* dorsal end of uterus, *po* posterior oviduct

To elucidate where and when the crosslinking occurs, histological properties of LNSP1, LNSP2 and TG were investigated. As expected, positive signals of both *LNSP1* and *LNSP2* were mainly detected over the entire area of the accessory gland with reduced expression being detected in the uterus (Fig. 1a, b; for the negative control (sense probe) images, see additional file 3: Fig. S2). Although two morphologically distinguishable lobes were present in each side accessory gland, no apparent differences in the signals of *LNSP1* or *LNSP2* were observed between the two lobes, suggesting that both LNSP1 and LNSP2 are expressed in the same tissues. In contrast, *TG* signal was exclusively detected in a highly localized area around the opening of the posterior oviduct (Fig. 1c; see Additional file 3: Fig. S2 for the negative control). These results strongly demonstrated that large amounts of both LNSP1 and LNSP2 are commonly expressed in the accessory glands and uterus without any histological separation, whereas TG expression is limited to a very narrow area around the opening of the posterior oviduct.

### Microscopic observation of oviposition process

**Fig. 2:**
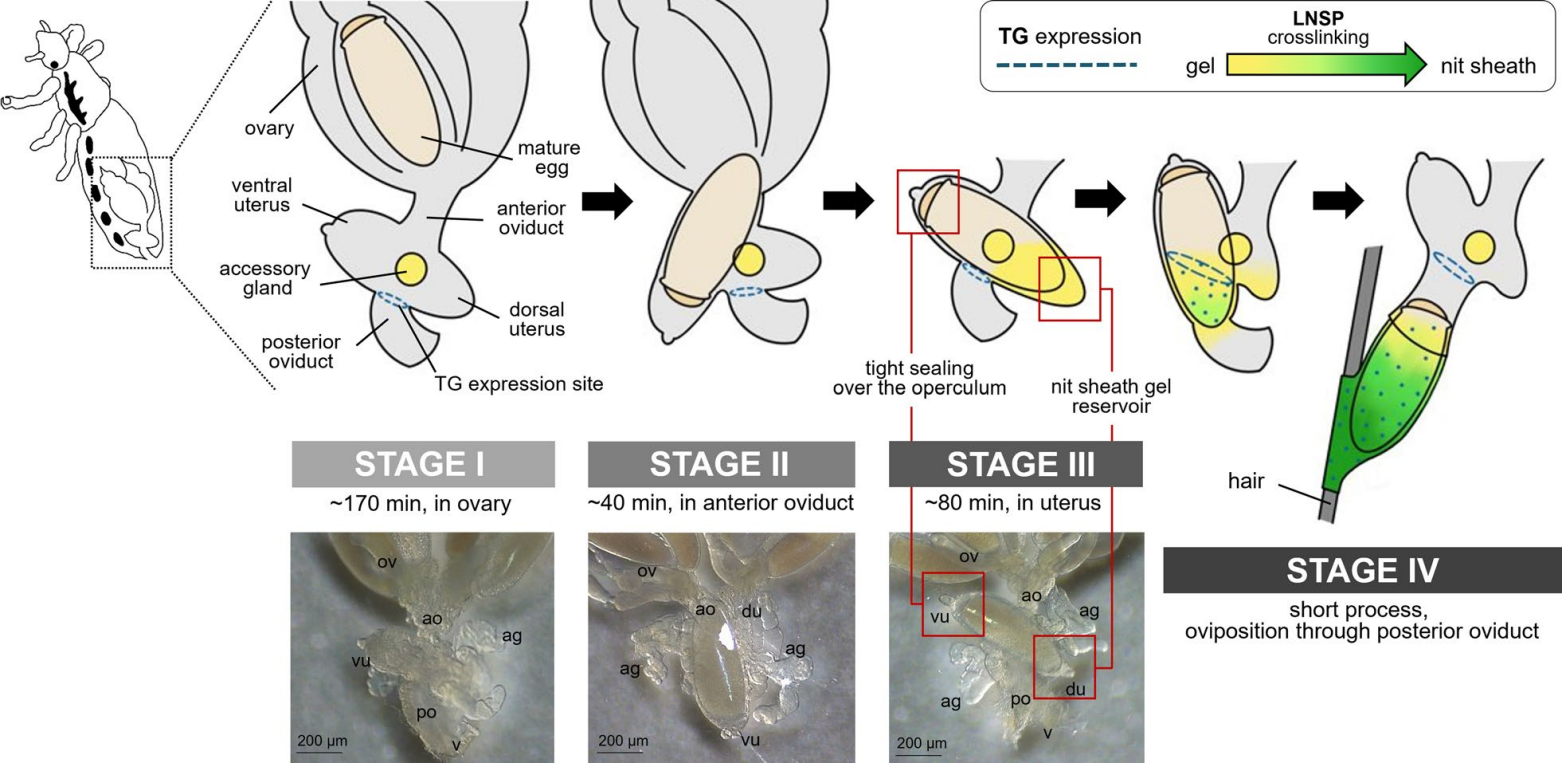
Schematic diagram of the oviposition processes of head louse females. For better view, accessory gland was graphically removed, and connecting area of accessory gland was marked with a yellow circle. Stage I, all mature eggs are still inside the ovary (~ 170 min); stage II, mature eggs are ovulated into the anterior oviduct (~ 40 min). During ovulation, a mature egg first moves to the other side of lateral oviduct, thereby changing its position to upside down, and then to the anterior oviduct; stage III, mature eggs stay inside the uterus and are coated with nit sheath gel materials in the dorsal end of the uterus (~ 80 min); stage IV, coated eggs move to the posterior oviduct where crosslinking begins. Actual images corresponding to each stage were provided in the bottom row. ov, ovary; ao, anterior oviduct; vu, ventral end of uterus; du, dorsal end of uterus; ag, accessory gland; po, posterior oviduct; v, vulva

**Fig. 3:**
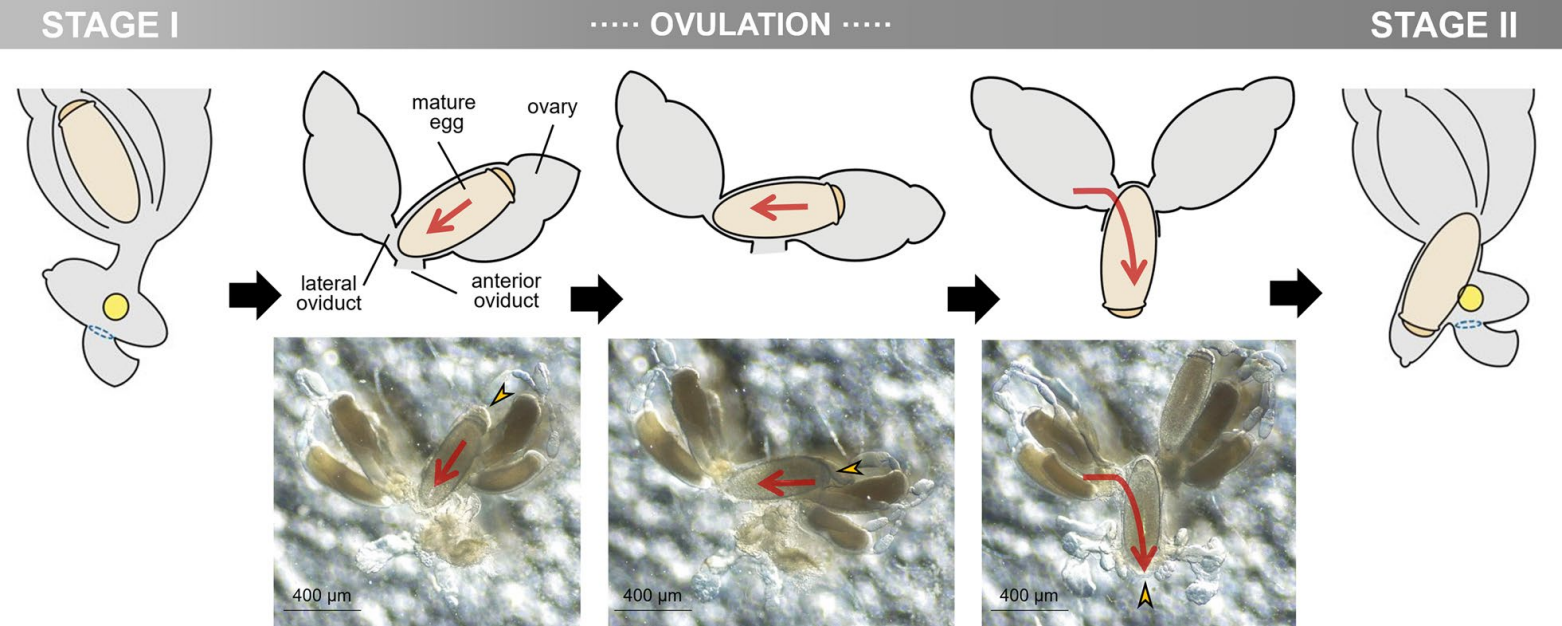
Detailed ovulation processes during the oviposition stages I and II. A mature egg first moves to the other side of lateral oviduct, thereby changing its position to upside down, and then to the anterior oviduct. The red arrows indicate the movement direction of mature eggs inside the oviduct during ovulation. The yellow arrowheads indicate operculum of an egg

Detailed microscopic observations revealed that the oviposition processes could be divided into four stages (Fig. 2): stage I, developing eggs are housed inside the ovary with its operculum side being located toward the head; stage II, mature eggs are ovulated into the anterior oviduct. During ovulation, a mature egg first moves to the other side of the lateral oviduct, thereby changing its position to upside down, and then then enters into the ventral end of the uterus with its operculum side moving first (Fig. 3); stage III, mature eggs move to and stay inside the uterus following ovulation and are coated with nit sheath gel. Once aligned inside the uterus, the mature egg is redirected so that its operculum side is toward the head again and its pointed bottom end is positioned toward the vagina; stage IV, coated eggs move to the posterior oviduct where crosslinking begins. Based on the approximation that one cycle of oviposition takes ~ 290 min (see Examination of oviposition process section), pre-ovulation stage I, stage II and stage III were estimated to take ~ 170 min, ~ 40 min and ~ 80 min, respectively. The last oviposition step (stage IV) was processed very quickly as described previously [3].

**Fig. 4:**
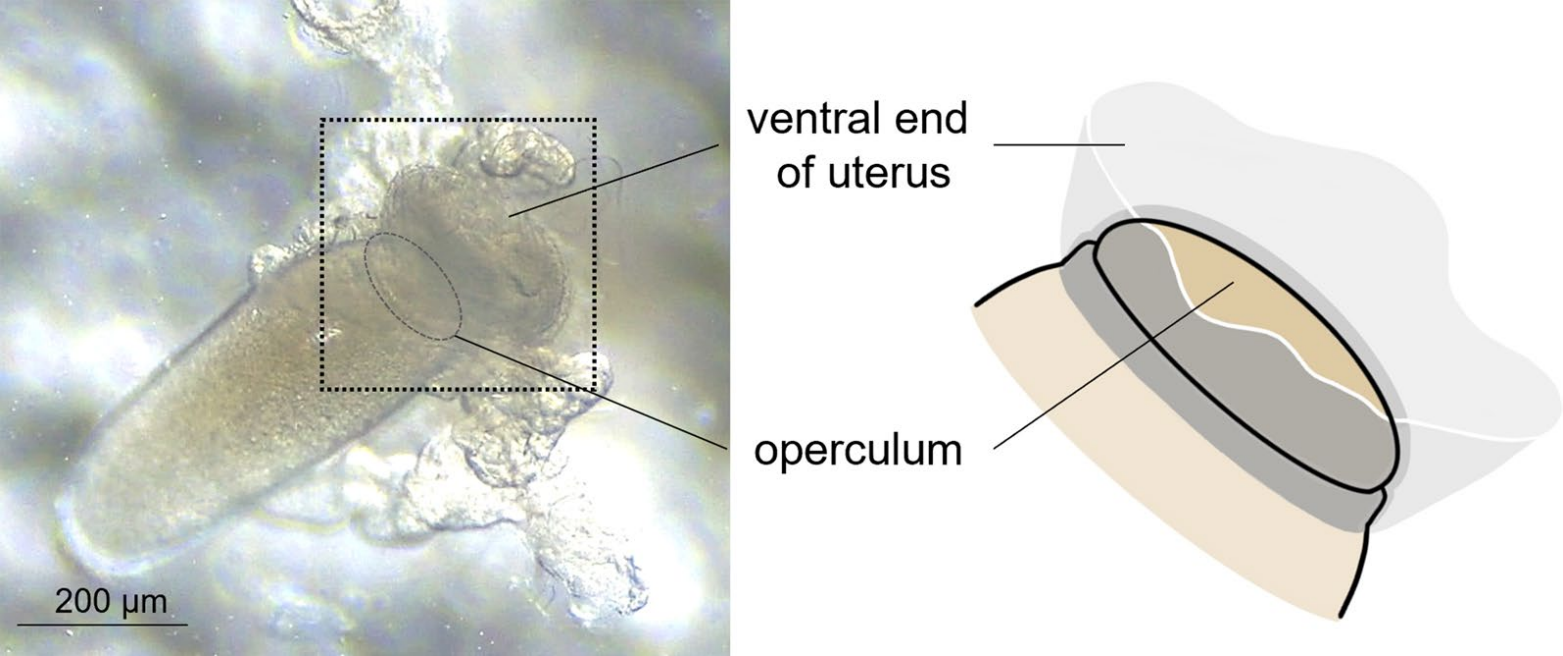
Image of a mature egg with its operculum attached to the ventral end of the uterus. The operculum stays attached to the ventral end of the uterus even after surgically removing the dorsal part of the uterus. The ventral uterus turns inside out in this picture

**Fig. 5:**
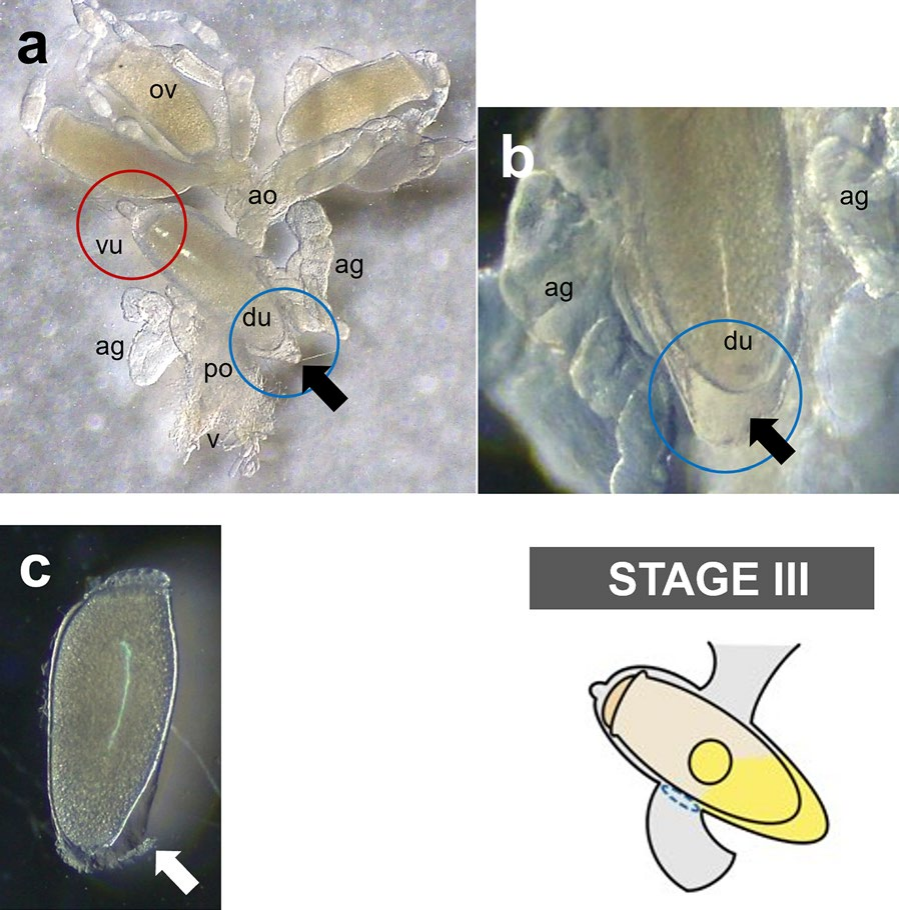
Reproductive system of a female head louse during oviposition. **a, b** A mature egg is aligned inside the uterus with the operculum being tightly covered by the ventral end of the uterus (see red circle) and bottom side covered by the dorsal end of the uterus (see blue circle) during the stage III. Black arrows indicate the nit sheath gel reservoir in the dorsal end of the uterus. ov, ovary; ao, anterior oviduct; vu, ventral end of uterus; du, dorsal end of uterus; ag, accessory gland; po, posterior oviduct; v, vulva. **c** An egg dissected out from uterus. A white arrow indicates nit sheath gel coated on the egg

Interestingly, during stage III, the mature egg is precisely aligned inside the uterus, with the operculum (top side) being tightly held by the ventral end of the uterus and the bottom side covered by the dorsal end of the uterus (see stage III in Fig. 2). Once held by the ventral end of the uterus, a tight connection appeared formed around the circumference of the operculum as judged from the observation that the operculum stayed attached to the ventral end of the uterus even after surgically removing the dorsal part of the uterus (Fig. 4). Thus, like a tight swimming cap, the ventral end of the uterus strongly holds the rim of the operculum, thereby physically protecting the operculum area from being coated by nit sheath gel and subsequent crosslinking. In contrast, nit sheath gel was observed inside the dorsal end of the uterus, suggesting that the dorsal area of the uterus may function as a reservoir for the secreted LNSP1 and LNSP2 (Fig. 5a, b). When an egg was dissected out from the uterus, viscous nit sheath gel was isolated, being coated mostly over the bottom of the egg (Fig. 5c). This finding suggested that the nit sheath gel is adhesive to the surface of the egg but not to the inner wall of the uterus.

## Discussion

In the fruit fly, *Drosophila melanogaster*, TG is expressed in hemocytes in either secreted or cytosolic form [8]. The secreted form of TG, which is equivalent to the coagulation factor XIIIa in human blood, is involved in the hemolymph coagulation and hardening by crosslinking various clot components, thereby serving as an immune component [9, 10]. The human head louse TG involved in the crosslinking of nit sheath proteins, however, was expressed only at the opening of the posterior oviduct as determined by in situ hybridization (Fig. 1c). Further molecular characterization revealed that TG has neither signal sequences at the N-terminus nor a putative anchoring site at the C-terminal region. The apparent inhibition of nit sheath solidification by hair-coated GGsTop, an irreversible inhibitor of TG [6], however, suggests that TG is secreted into the lumen of oviduct likely via a non-classical secretion pathway [11].

Interestingly, the TG expression site was spatially separated from the ventral end of the uterus, which tightly holds the operculum side of the mature egg during oviposition, thus likely protecting it from contacting the nit sheath gel (Fig. 2). As both LNSP1 and LNSP2 are expressed in the same tissues of the accessory gland and uterus without any physical separation (Fig. 1), confining the crosslinking site to the opening of the posterior oviduct is critical to avoid uncontrolled crosslinking inside the lumen of the accessory gland or reproductive system. Crosslinking would be initiated from the bottom of the egg toward the top during the oviposition process, thereby producing eggs with the operculum side uncoated with nit sheath gel only when the accessory gland secretion containing LNSP1 and LNSP2 is mixed with TG at the entrance of the posterior oviduct (see stage IV in Fig. 2). The sophisticated mechanism for the precise alignment of a mature egg inside the uterus was revealed, for the first time to our knowledge, through detailed microscopic observation in this study: a mature egg first changes its position to upside down to make the operculum side first enter the ventral end of the uterus in stage II, and then it is repositioned so that its pointed bottom end enters the opening of the posterior oviduct, where crosslinking is initiated. In addition to the tight physical protection of the operculum by the ventral end of the uterus, additional possible mechanisms may include the presence of protective substances and/or nanostructure inside the ventral side of the uterus and/or over the surface of the operculum, which can impede the nit sheath gel coating and crosslinking. When gel secretion containing TG leaves the vulva, further TG-mediated crosslinking of nit sheath gel continues until complete solidification, as demonstrated by the finding that GGsTop (a TG inhibitor)-coated hair retarded the solidification process of deposited eggs [6]. Following oviposition, the atmospheric oxygen may function as an additional triggering factor for the completion of the solidification process [4]. Nevertheless, considering the observation that nit sheath gel was solidified inside the uterus, thus blocking oviposition when *LNSP2* was knocked down via RNAi [6], the solidification by atmospheric oxygen may not be an essential requirement.

## Conclusions

LNSP1 and LNSP2, major constituents of the nit sheath expressed over the entire area of accessory glands and uterus, become solidified by TG-mediated crosslinking, which occurs at the confined area around the opening of the posterior oviduct. Physical separation of the crosslinking site allows selective crosslinking over only the lower part of the egg. The precise coordination of the oviposition processes and nit sheath gel secretion and crosslinking is crucial for both female and embryo survival; this step can be exploited as a potential target for louse control by disturbing the controlled formation of the egg sheath. In addition, the novel information on the nit sheath-forming and egg-laying processes in human lice can be expanded to other insect species, facilitating the understanding of hidden functions of the extra egg sheath.

## Supplementary Information

**Additional file 1: Table S1**. Primers used in this study.

**Additional file 2: Fig. S1.** Female reproductive system of human head louse.

**Additional file 3: Fig. S1.** Female reproductive system of human head louse.

## Data Availability

Not applicable.

## Abbreviations

LNSP: Louse nit sheath protein
TG: Transglutaminase
RNAi: RNA interference
PBS: Phosphate-buffered saline
SSC: Saline sodium citrate

## References

[1] Clark JM (2009). Determination, mechanism and monitoring of knockdown resistance in permethrin-resistant human head lice, *Pediculus humanus capitis*. J Asia-Paci Entomol.

[2] Ferris GF. The sucking lice. Mem Pacif Coast Ent Soc. 1951 1.

[3] Nuttall GH (1919). The biology of *Pediculus humanus*. Parasitology.

[4] Carter DG (1990). Insect egg glue: an investigation of the nature and secretion of insect egg glues, with special reference to the human louse, *Pediculus humanus* and the cabbage white butterfly.

[5] Park JK, Han YJ, Lee JH, Joo S-W, Kim JH, Lee SH (2019). Characterization of the human head louse nit sheath reveals proteins with adhesive property that show no resemblance to known proteins. Sci Rep.

[6] Kim JH, Lee DE, Park S, Clark JM, Lee SH (2021). Characterization of nit sheath protein functions and transglutaminase-mediated cross-linking in the human head louse, *Pediculus humanus capitis*. Parasit Vectors.

[7] Yoon KS, Strycharz JP, Gao J-R, Takano-Lee M, Edman JD, Clark JM (2006). An improved in vitro rearing system for the human head louse allows the determination of resistance to formulated pediculicides. Pestic Biochem Physiol.

[8] Shibata T, Hadano J, Kawasaki D, Dong X, Kawabata Si (2017). Drosophila TG-A transglutaminase is secreted via an unconventional Golgi-independent mechanism involving exosomes and two types of fatty acylations. J Biol Chem.

[9] Wang Z, Wilhelmsson C, Hyrsl P, Loof TG, Dobes P, Klupp M (2010). Pathogen entrapment by transglutaminase—a conserved early innate immune mechanism. PLoS Pathog.

[10] Shibata T, Kawabata S-i (2018). Pluripotency and a secretion mechanism of *Drosophila* transglutaminase. J Biochem.

[11] Nickel W (2003). The mystery of nonclassical protein secretion: a current view on cargo proteins and potential export routes. Eur J Biochem.

