# Supplementary material for: Histological confinement of transglutaminase-mediated nit sheath crosslinking is essential for proper oviposition and egg coating in the human head louse, Pediculus humanus capitis
Source: Parasit Vectors. 2023 Mar 9;16:93. doi: 10.1186/s13071-023-05720-5 (PMC9997029; doi:10.1186/s13071-023-05720-5)
Supplement: Supplementary file 1 — Additional file 1: Table S1. Primers used in this study. [file 13071_2023_5720_MOESM1_ESM.docx]

**Table S1.** Primers used in this study

| Purpose | Gene | Sequence (5’ to 3’) | | Product size (bp) |
| --- | --- | --- | --- | --- |
| Probe | *LNSP1* | Forward  Reverse | ACTTTTCGCCGCTGGATTCG  GTTCTTGAACGACGGGGGAA | 518 |
|  | *LNSP2* | Forward  Reverse | TTCGTACCCCATGCACGGAT  CAACTCTTCTGCTTCCGACG | 420 |
|  | *TG* | Forward  Reverse | GGGATGCTGCAGTACACGAA  ATAATGGCGACGGATCCGGT | 511 |
