# Supplementary figures and images for: Histological confinement of transglutaminase-mediated nit sheath crosslinking is essential for proper oviposition and egg coating in the human head louse, Pediculus humanus capitis
Source: Parasit Vectors. 2023 Mar 9;16:93. doi: 10.1186/s13071-023-05720-5 (PMC9997029; doi:10.1186/s13071-023-05720-5)

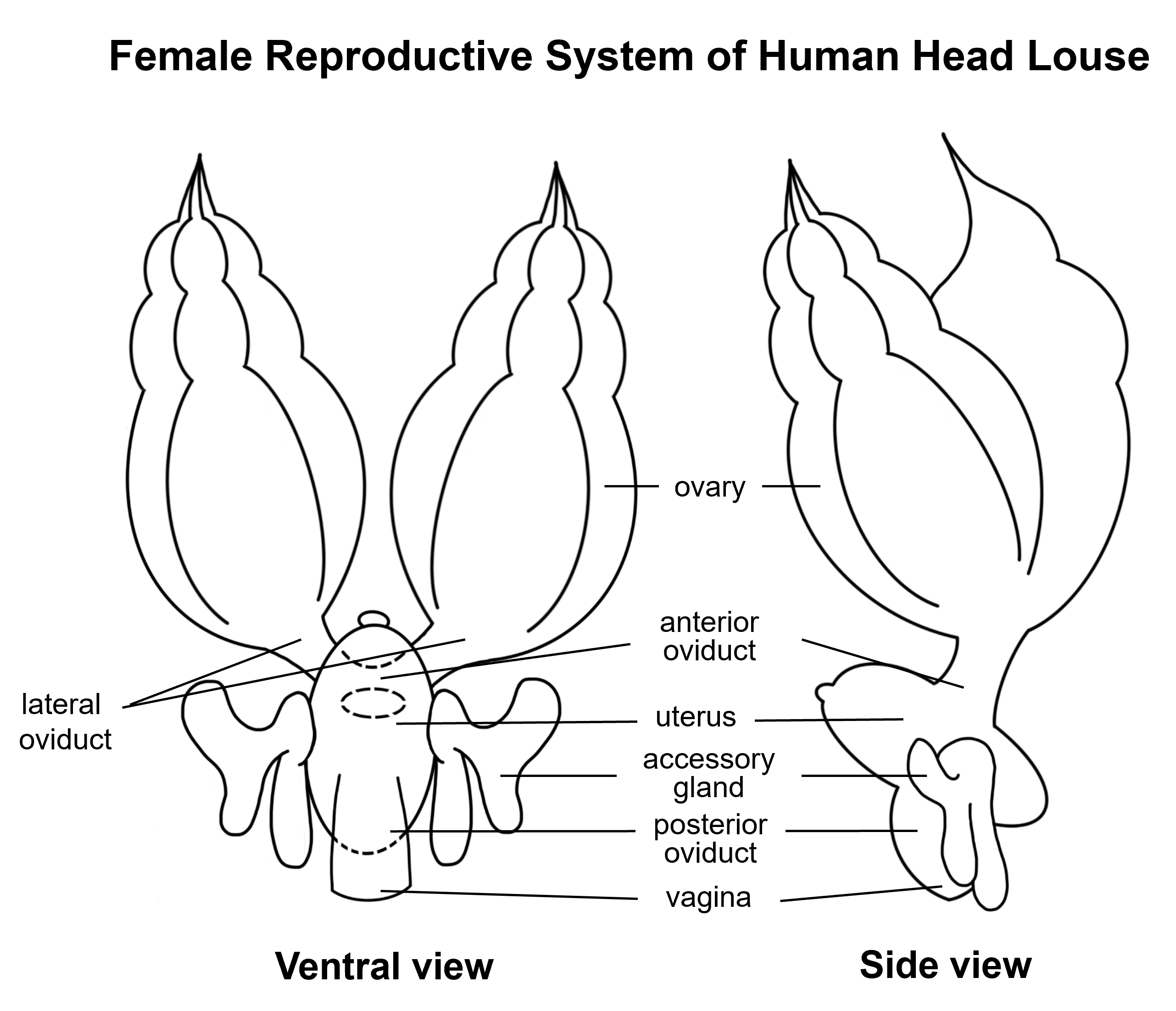

Supplement: Supplementary file 2 — Additional file 2: Fig. S1. Female reproductive system of human head louse. [file 13071_2023_5720_MOESM2_ESM.tif]

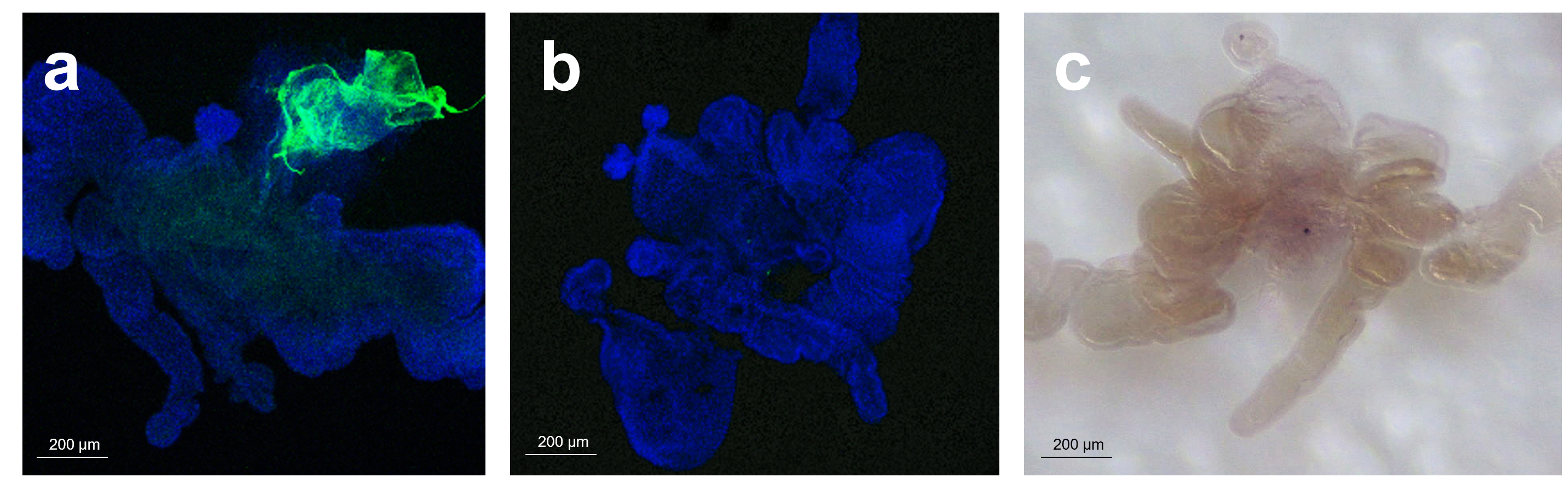

Supplement: Supplementary file 3 — Additional file 3: Fig. S1. Female reproductive system of human head louse. [file 13071_2023_5720_MOESM3_ESM.tif]
